# Modulation of BIN2 kinase activity by HY5 controls hypocotyl elongation in the light

**DOI:** 10.1038/s41467-020-15394-7

**Published:** 2020-03-27

**Authors:** Jian Li, William Terzaghi, Yanyan Gong, Congran Li, Jun-Jie Ling, Yangyang Fan, Nanxun Qin, Xinqi Gong, Danmeng Zhu, Xing Wang Deng

**Affiliations:** 1 State Key Laboratory of Protein and Plant Gene Research, School of Advanced Agricultural Sciences and School of Life Sciences, Peking-Tsinghua Center for Life Sciences, Peking University, 100871 Beijing, China; 2 Institute of Plant and Food Science, Department of Biology, Southern University of Science and Technology, 518055 Shenzhen, China; 3 Department of Biology, Wilkes University, Wilkes-Barre, PA 18766 USA; 4 Institute for Mathematical Sciences, Renmin University of China, 100872 Beijing, China

**Keywords:** Plant sciences, Light responses, Brassinosteroid, Plant signalling

## Abstract

ELONGATED HYPOCOTYL 5 (HY5), a basic domain/leucine zipper (bZIP) transcription factor, acts as a master regulator of transcription to promote photomorphogenesis. At present, it’s unclear whether HY5 uses additional mechanisms to inhibit hypocotyl elongation. Here, we demonstrate that HY5 enhances the activity of GSK3-like kinase BRASSINOSTEROID-INSENSITIVE 2 (BIN2), a key repressor of brassinosteroid signaling, to repress hypocotyl elongation. We show that HY5 physically interacts with and genetically acts through BIN2 to inhibit hypocotyl elongation. The interaction of HY5 with BIN2 enhances its kinase activity possibly by the promotion of BIN2 Tyr^200^ autophosphorylation, and subsequently represses the accumulation of the transcription factor BRASSINAZOLE-RESISTANT 1 (BZR1). Leu^137^ of HY5 is found to be important for the HY5-BIN2 interaction and HY5-mediated regulation of BIN2 activity, without affecting the transcriptional activity of HY5. HY5 levels increase with light intensity, which gradually enhances BIN2 activity. Thus, our work reveals an additional way in which HY5 promotes photomorphogenesis, and provides an insight into the regulation of GSK3 activity.

## Abstract (web summary)

HY5 is a bZIP transcription factor and master regulator of photomorphogenesis in plants. Here, the authors show that in addition to regulating transcription, HY5 promotes the activity of the GSK3-like kinase BIN2 thus negatively regulating hypocotyl elongation by suppressing brassinosteroid signaling.

## Introduction

Light is not only the ultimate source of energy but also one of the most important environmental signals for plants. Light plays a key role in the morphogenesis of *Arabidopsis* seedlings^1^. One of the most remarkable events in light-controlled morphogenesis is hypocotyl elongation. While germinating under soil without light, seedlings accelerate hypocotyl elongation in order to reach the light. Upon reaching the light, hypocotyl elongation is finely controlled to match the ambient light intensity^2,3^.

The light signal is transduced from various photoreceptors to downstream transcription factors, which regulate hypocotyl elongation by modulating transcription. HY5 is one of these light-responsive transcription factors that play a key role in repressing seedling hypocotyl elongation^4,5^. It belongs to the basic leucine zipper (bZIP) family of transcription factors^6^. The C-terminal domain of HY5 harbors a basic region that binds DNA and a leucine zipper for dimerization^6,7^. HY5 regulates transcription by directly binding to *cis*-acting elements in promoters, including the G-box, Z-box, and others^8^. Genome-wide ChIP-chip analyses demonstrated that HY5 directly bound the promoters of nearly 3000 genes and regulated the transcription of one-third of all genes^8^. The transcriptional activity of HY5 is influenced by its physical interaction with other transcription factors. HYH, the homolog of HY5, can form heterodimers with HY5 to promote HY5 activity^9,10^. In contrast, the B-box-containing protein BBX28 can repress the transcriptional activity of HY5 by direct interaction^11^. HY5 protein is degraded by COP1-mediated ubiquitination in the dark^3,12^. The COP1-HY5 module represents the core regulatory mechanism that regulates seedling morphogenesis, including hypocotyl elongation. Many other proteins, such as CALMODULIN7 (CAM7), WRKY DNA-BINDING PROTEIN 36 (WRKY36), and SHI-RELATED SEQUENCE5 (SRS5), regulate hypocotyl elongation by modulating the transcription of HY5^13–15^, suggesting a central role of HY5 in the inhibition of hypocotyl elongation.

It has long been recognized that brassinosteroids (BRs) are also involved in light-controlled hypocotyl elongation^16^. BR-deficient mutants show shorter hypocotyls than wild type in the dark, suggesting BR promotes hypocotyl elongation^16,17^. As the key transcription factor responding to BR, BZR1 can promote hypocotyl elongation through its transcriptional activity^18^. The stability and DNA-binding activity of BZR1 protein is negatively regulated by BIN2, the *Arabidopsis* ortholog of human GLYCOGEN SYNTHASE KINASE 3 (GSK3) kinase. BIN2 blocks DNA binding and promotes degradation of BZR1 through phosphorylation^19,20^, which results in the inhibition of hypocotyl elongation.

The dwarf phenotype of *bri1-5*, a weak mutant of BR receptor BR INSENSITIVE 1 (BRI1), can be partially rescued by crossing it with mutants lacking PHYTOCHROME B (phyB)^21^, suggesting complicated regulatory interactions between light and BR signals. Several reports studying the molecular interactions involved in the cross-talk between light and BR signaling found that physical association of transcription factors acting in the two signaling pathways played a critical role in modulating hypocotyl elongation. Notably, BZR1 can directly interact with PHYTOCHROME-INTERACTING FACTOR 4 (PIF4)^22^. They share nearly 2000 common target genes, and act interdependently in promoting hypocotyl elongation. BZR1 can also directly regulate the level of GATA2 mRNA^23^, a transcription factor repressing hypocotyl elongation acting downstream of both light and BR signals. Recent studies revealed that the photoreceptors UV RESISTANCE LOCUS 8 (UVR8) and CRYPTOCHROME 1 (CRY1) could interact with BES1 in UV-B or blue light, respectively, to inhibit its DNA-binding ability and thereby inhibit hypocotyl elongation^24,25^. CRY1 could also regulate BZR1 by repressing its transcriptional activity and promoting its phosphorylation by BIN2^26^. However, as the core factor in light signaling, the mechanism whereby HY5 regulates hypocotyl elongation in response to the cross-talk between light and BR signals remains unclear. In addition, a previous study showed that overexpression of HY5 reduced BZR1 protein levels in the light but the mechanism remains unknown^27^.

Here we show that in addition to its function as a transcription factor, HY5 can enhance BIN2 kinase activity through physical interaction, thereby promoting BIN2-mediated phosphorylation and degradation of BZR1, which represses BR-mediated hypocotyl elongation in the light.

## Results

### HY5 physically interacts with BIN2

**Fig. 1:**
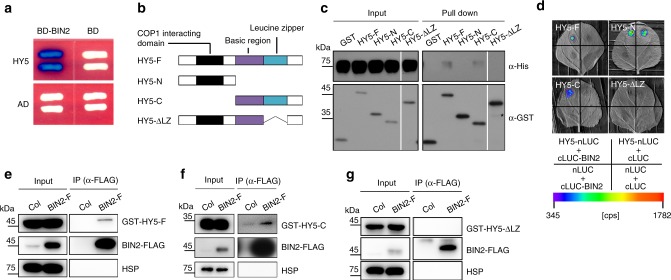
HY5 interacts with BIN2. **a** HY5 interacts with BIN2 in yeast. Full-length HY5 was fused with AD. **b** Schematic representation of HY5. HY5-F, full-length HY5; HY5-N, aa 1–77; HY5-C, aa 78–168; HY5-ΔLZ, HY5 lacking leucine zipper region (LZ, aa 115–147). **c** In vitro pull-down assay showing the interaction between HY5 and BIN2. Full-length and truncated HY5 were fused with the GST-tag; BIN2 was fused with the His-tag. Asterisk indicates a non-specific band. **d** LCI assay showing the interaction between HY5 and BIN2 in *N. tabacum* leaves. nLUC, the vector containing the N-terminal fragment of firefly luciferase. cLUC, the vector containing the C-terminal fragment of firefly luciferase. Full-length and truncated HY5 was fused with nLUC and BIN2 was fused with cLUC. The *N. tabacum* leaves were infiltrated with the indicated combinations. cps, counts per second. **e**–**g** Co-IP assays showing HY5 associated with BIN2. HSP was used as a loading control. Source data are provided as a source data file.

To investigate the cross-talk between light and BR signaling, we performed yeast two-hybrid analyses and found that BIN2, the key negative regulator of BR signaling, interacted with HY5 (Fig. 1a). Likewise, the two homologs of BIN2, BIN2-Like1 (BIL1) and BIN2-Like2 (BIL2), also interacted with HY5 in yeast (Supplementary Fig. 1a). To map the subdomain of HY5 required for BIN2 interaction, we first divided the full-length HY5 CDS into N- and C-terminal domains and tested their interactions with His-BIN2 by in vitro pull-down assays. We found that the C-terminal domain of HY5 was sufficient for it to interact with BIN2 (Fig. 1b, c). Deletion of the leucine zipper (LZ) in the C-terminal domain of HY5 abolished the interaction between HY5 and BIN2 in vitro. Next, the in vivo interactions between full-length or truncated HY5 with BIN2 were confirmed using firefly luciferase complementation imaging (LCI) (Fig. 1d, Supplementary Fig. 1b) and co-immunoprecipitation (Co-IP) assays (Fig. 1e–g). Together, these experiments showed that the C-terminal domain of HY5, especially the LZ motif, was essential for interaction with BIN2.

### HY5 genetically interacts with BIN2 and BZR1

**Fig. 2:**
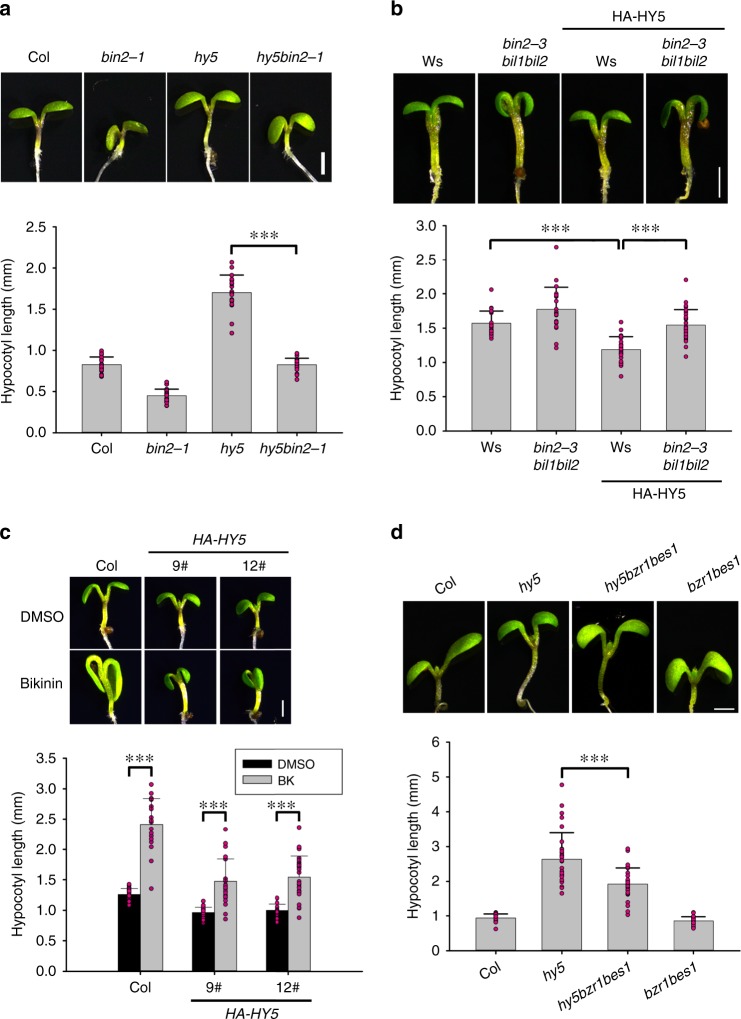
HY5 genetically interacts with BIN2 and BZR1 to inhibit hypocotyl elongation in the light. **a** Hypocotyl lengths of Col, *bin2-1, hy5*, and *hy5bin2-1* mutant seedlings. **b** Hypocotyl lengths of lines overexpressing HY5 in Ws and *bin2-3bil1bil2* backgrounds. *bin2-3bil1bil2*, null mutant of *BIN2* and its closest homologs, *BIL1* and *BIL2*. *HA-HY5*, transgenic seedlings overexpressing 3×*HA*-tagged *HY5*. **c** Hypocotyl lengths of *HA-HY5* line seedlings treated with 30 μM bikinin (BK), an inhibitor of *Arabidopsis* GSK3-like kinases. **d** Hypocotyl lengths of light-grown seedlings of *hy5bzr1bes1* and *bzr1bes1*. *BZR1* and *BES1* double mutants were generated by CRISPR/Cas9. Seedlings were grown for 6 days under continuous white light (cWL). Seedlings were grown under 20 W m^−2^ (**a**, **d**) or 10 W m^−2^ (**b**, **c**) light intensity, respectively. Error bars represent standard deviation (SD), *n* ≥ 20. Asterisks indicate the *P* value of two-tailed Student’s *t*-test; ***P < 0.001. Experiments were performed three times with similar results. Bar = 1 mm. Source data are provided as a source data file.

Next, we investigated whether HY5 could genetically interact with BIN2 by crossing *hy5* into *bin2-1*, a gain-of-function mutant of *BIN2*^28^. We found that the hypocotyls of double mutant *hy5bin2-1* seedlings were the same length as those of *bin2-1* when grown in the dark (Supplementary Fig. 2), indicating that HY5 had no effect on the short hypocotyl phenotype of *bin2-1* in the dark. However, *hy5bin2-1* hypocotyls were dramatically shorter than those of *hy5* grown under continuous white light (cWL) (Fig. 2a). Likewise, seedlings overexpressing *HY5* (*HA-HY5*) in *bin2-3bil1bil2*, the triple mutant of *BIN2* and its two homologs, exhibited longer hypocotyls than those of *HA-HY5* in cWL (Fig. 2b), suggesting that HY5 and BIN2 genetically interacted to regulate hypocotyl elongation. To further evaluate the contribution of BR signaling to the short hypocotyls of light-grown *HA-HY5* seedlings, we applied bikinin (BK)^29^, a specific inhibitor of GSK3-like kinases including BIN2, to WT and two independent *HA-HY5* lines. As shown in Fig. 2c, hypocotyls of *HA-HY5* lines significantly elongated after BK treatment. In addition, the HY5 protein stability was not influenced by BIN2 in the light (Supplementary Fig. 3). Taken together, these results suggest that BIN2 and its homologs act epistatically to HY5 in the control of hypocotyl elongation.

It was previously reported that a key step in BR signaling is the phosphorylation of BZR1 by BIN2 to mark it for degradation to control hypocotyl elongation^30^. We therefore tested whether BZR1 was involved in the HY5-mediated inhibition of hypocotyl elongation. We used the CRISPR/Cas9 genome editing system to generate *bzr1bes1*^31^, a double mutant knocking out both *BZR1* and its homolog *BES1*. We then crossed *bzr1bes1* into *hy5* mutants, and found that the hypocotyls of the triple mutants were shorter than those of *hy5* (Fig. 2d). This suggested that BZR1 acts downstream of HY5 to antagonize its function in controlling hypocotyl elongation in the light.

### HY5 represses BZR1 accumulation in a BIN2-dependent manner

**Fig. 3:**
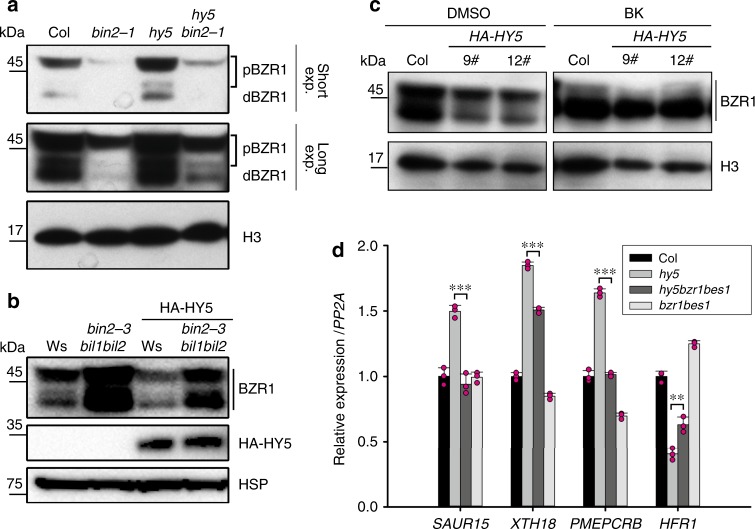
HY5 represses the accumulation of BZR1 in a BIN2-dependent manner in the light. **a** Protein levels and phosphorylation status of BZR1 in Col and the indicated mutants. *bin2-1,* gain-of-function mutant of *BIN2*. Short exp., short exposure. Long exp., long exposure. pBZR1, the phosphorylated form of BZR1; dBZR1, the dephosphorylated form of BZR1. H3 was used as a loading control. **b** BZR1 protein levels in HA-HY5/Ws and HA-HY5/*bin2-3bil1bil2* respectively. HSP was used as a loading control. **c** BZR1 protein levels in *HA-HY5* lines treated with 10 μM BK. H3 was used as a loading control. **d** Relative expression of genes regulated by BZR1 in Col and the indicated mutants. The relative expression levels were normalized to *PP2A*. Error bars represent SD, *n* = 3. Asterisks indicate the *P* value of two-tailed Student’s *t*-test; **P < 0.01; ***P < 0.001. RT-qPCR was performed three times with similar results. Source data are provided as a source data file.

Previous reports have suggested that HY5 and BIN2 have similar effects on BZR1 protein accumulation^20,27^. To gain further insight into their interplay, we checked BZR1 mRNA and protein levels in the *hy5* and *HA-HY5* lines. Our RT-qPCR results showed that the levels of BZR1 transcripts in *hy5* and *HA-HY5* were similar to those in wild type (Supplementary Fig. 4a), suggesting that HY5 did not affect the transcription of *BZR1*. However, the level of BZR1 protein was significantly higher in *hy5* than in wild type, and this elevated level was repressed by the introduction of *bin2-1* (Fig. 3a). This result suggested that HY5 might regulate the stability of BZR1 in a BIN2-dependent manner. In agreement with previous observations^27^, the decline in BZR1 protein level mediated by overexpression of HY5 was dependent on HY5 dosage (Supplementary Fig. 4b). Moreover, HY5 promoted the degradation of BZR1 via the 26S proteasome (Supplementary Fig. 4c). In addition, the reduced expression of BZR1 in *HA-HY5* was restored when *HA-HY5* was crossed into the *bin2-3bil1bil2* triple mutant (Fig. 3b). Consistent with this result, the application of BK to the two independent *HA-HY5* transgenic lines restored BZR1 protein levels to those of wild type (Fig. 3c). These results, taken together, indicate that in the light HY5 negatively regulates BZR1 accumulation in a BIN2-dependent manner.

Next, to confirm whether BZR1 contributed to the altered gene expression in *hy5*, we selected several BZR1-regulated genes involved in hypocotyl elongation and measured their expression in *hy5* and *hy5bzr1bes1* by quantitative real-time PCR (qPCR). Our results showed that the altered expression of these genes in *hy5* could be partially restored by the absence of BZR1 and BES1 (Fig. 3d). This indicated that part of the way in which HY5 regulates expression of genes controlling hypocotyl elongation is by destabilizing BZR1.

### HY5 interaction with BIN2 enhances BIN2 kinase activity

**Fig. 4:**
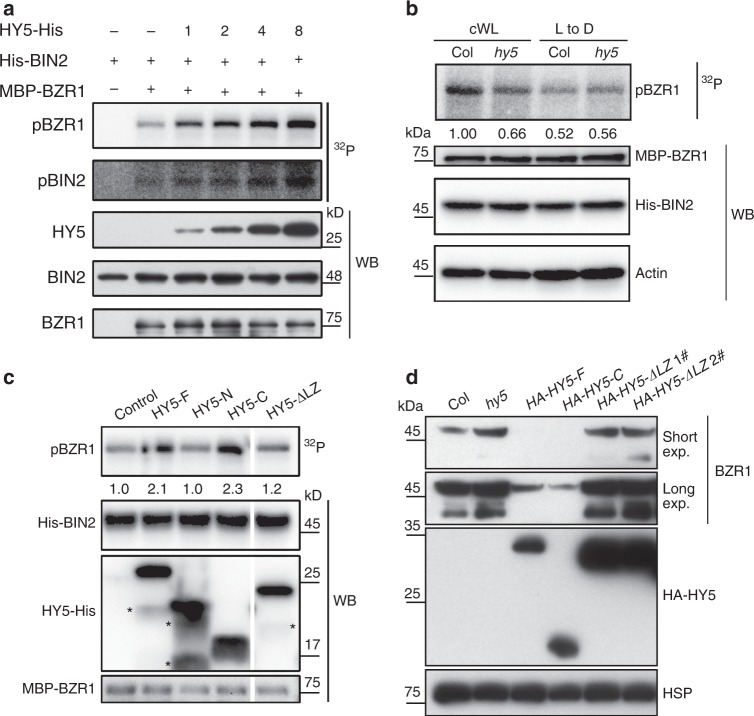
HY5 enhances BIN2-mediated phosphorylation of BZR1 through its C-terminal domain. **a** In vitro kinase assay showing HY5 enhanced kinase activity of BIN2. MBP-BZR1 was used as substrate for BIN2. 1, 2, 4, 8 indicate the mole ratios of HY5-His versus His-BIN2. pBZR1, phosphorylated BZR1; pBIN2, auto-phosphorylated BIN2. ^32^P, autoradiography of [γ-^32^P] ATP-labeled proteins. WB, western blot of proteins used in the kinase assay. **b** Cell-free kinase assay showing BIN2-mediated phosphorylation of BZR1 in the total lysates of Col and *hy5*. Seedlings grown in cWL were grown for another day in cWL, or transferred to the dark (L to D) for 1 day. Actin was used as a loading control. **c** Mapping the region of HY5 that was essential for the promotion of BIN2 activity in vitro. Asterisks indicate non-specific bands. **d** BZR1 protein levels in transgenic plants that overexpressed full-length and truncated HY5 in the *hy5* background. Short exp., short exposure. Long exp., long exposure. HSP was used as a loading control. Numbers under lanes in **b** and **c** indicate relative band intensities that were quantified for each panel. Source data are provided as a source data file.

To further identify the mechanism by which HY5 regulated BIN2 in the light to destabilize BZR1, we first checked whether HY5 regulated the mRNA and/or protein levels of BIN2. As shown in Supplementary Fig. 5, both the mRNA and protein levels of BIN2 were similar in *hy5* and WT, suggesting that HY5 does not affect the expression of BIN2. Next we tested whether HY5 influenced the interaction of BIN2 with BZR1 by in vivo Co-IP assays. These showed that the amounts of BZR1 Co-IPed by BIN2-FLAG were similar in *hy5* and wild type (Supplementary Fig. 6), indicating that HY5 had no effect on the interaction between BIN2 and BZR1. This prompted us to test whether HY5 modulated the kinase activity of BIN2 to regulate the phosphorylation of BZR1. We therefore performed in vitro kinase assays with His-BIN2 and MBP-BZR1, and found that BIN2 directly phosphorylated BZR1 in our system (Fig. 4a). Interestingly, we found that the phosphorylation levels of BZR1 were positively correlated with the amounts of HY5-His added to the kinase reactions (Fig. 4a, Supplementary Fig. 7), suggesting a synergistic effect of HY5 on BZR1 phosphorylation by BIN2. In addition, the autophosphorylation level of BIN2 was also significantly increased (Fig. 4a), suggesting that HY5 upregulated BIN2 activity in a dosage-dependent manner. To confirm this effect of HY5 in vivo, we performed cell-free kinase assays using total lysates of wild type and *hy5* seedlings supplemented with equal amounts of His-BIN2 and MBP-BZR1. The phosphorylation levels of BZR1 were clearly higher in WT than in *hy5* extracts prepared from light-grown seedlings but not in the extracts prepared from the seedlings transferred from light to the dark (Fig. 4b). This suggests that the phosphorylation of BZR1 by BIN2 is enhanced in the presence of HY5 in the light.

To further confirm that the enhancement of BIN2-mediated BZR1 phosphorylation by HY5 is dependent on HY5-BIN2 interactions, we conducted in vitro kinase assays using derivatives of truncated recombinant HY5 proteins. Our data showed that adding either the full-length HY5 (HY5-F) or C-terminal domain of HY5 (HY5-C) could increase BIN2-mediated phosphorylation levels of BZR1 to about two-fold higher than the control (Fig. 4c). Furthermore, inhibition of HY5-BIN2 binding by expressing HY5 lacking the LZ region completely abolished its capacity to upregulate BIN2 activity (Fig. 4c). This emphasizes the importance of HY5-BIN2 interactions in HY5 modulation of BIN2 activity. Next, we generated transgenic plants overexpressing either HY5-F or HY5-C in the *hy5* mutant background. As expected, overexpression of HY5-C or HY5-F in the *hy5* background could suppress the over-accumulation of BZR1 in *hy5*, while overexpression of HY5-ΔLZ in *hy5* failed to inhibit the over-accumulation of BZR1 (Fig. 4d). These results support the notion that binding of HY5 via its LZ region to BIN2 is essential for enhancing BIN2 activity. This increases the phosphorylation and degradation of BZR1 in the light.

### HY5 L137D mutation reduces BIN2 activity

**Fig. 5:**
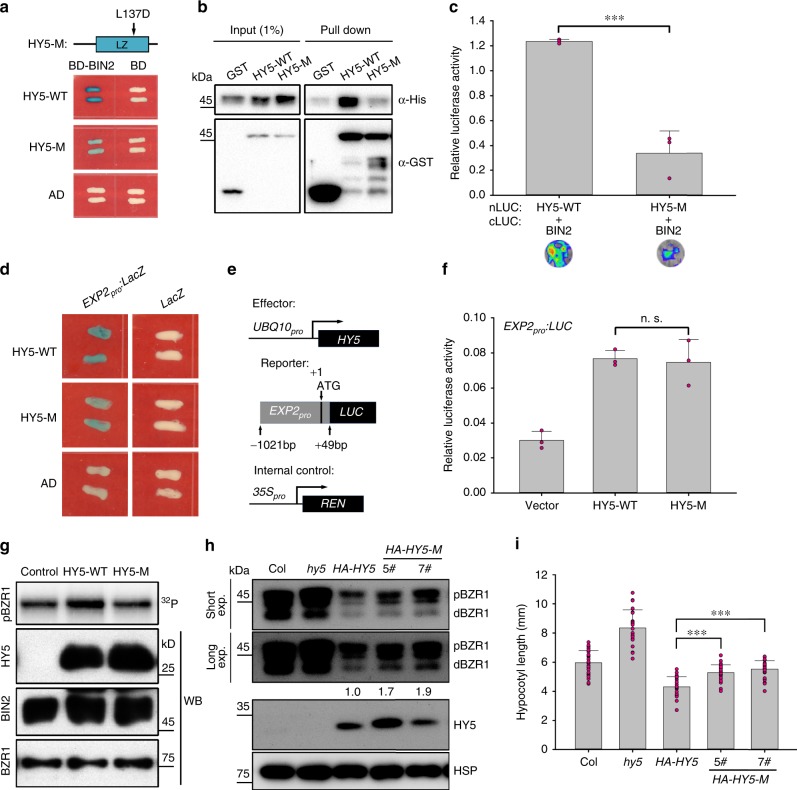
HY5 can regulate the kinase activity of BIN2 independently of its transcriptional activity. **a** The attenuated interaction between BIN2 and mutated HY5 (HY5-M) in yeast. WT wild type. **b** In vitro pull-down assay showing attenuated interaction between BIN2 and HY5-M. **c** LCI assay showing attenuated interaction between BIN2 and HY5-M in *N. tabacum* leaves. The values measuring the interaction between HY5 and BIN2 were obtained by subtracting the interaction between HY5 and the cLUC empty vector. Error bar represents SD, *n* = 3. Asterisks indicate the *P* value of two-tailed Student’s *t*-test; ***P < 0.001. Experiments were performed three times with similar results. **d** Yeast one-hybrid assay showing HY5-M could bind to the *EXP2* promoter in yeast similarly to HY5-WT. HY5 was fused with the activation domain (AD). The *EXP2* promoter was inserted upstream of the *LacZ* reporter gene. **e** Schematic representation of various constructs used in the transient transfection assay in *Arabidopsis* protoplasts. LUC, firefly luciferase. REN, renilla luciferase. **f** Relative *LUC* expression level driven by *EXP2*_*pro*_ in *Arabidopsis* protoplasts. The gene expression levels were quantified as the ratios of LUC/REN enzyme activities. Error bar represents SD of three independent transient transfections in protoplasts. n.s., not significant in two-tailed Student’s *t*-test. Experiments were performed three times with similar results. **g** In vitro kinase assay showing HY5-M could not enhance BIN2 activity. Control, no recombinant HY5 was added to the reaction. **h** The abundance of the dephosphorylated form of BZR1 in *HA-HY5-M* lines. Numbers below the bands indicated the relative band intensities of dBZR1. pBZR1, phosphorylation form of BZR1; dBZR1, dephosphorylation form of BZR1. *HA-HY5* or *HA-HY5-M* were overexpressed in the *hy5* mutant background. HSP was used as a loading control. **i** Hypocotyl lengths of HA-HY5/*hy5* and HA-HY5-M/*hy5*, respectively, in the light. Seedlings were grown for 6 days in the light. Error bars represent SD, *n* ≥ 20. Asterisks indicate the *P* value of two-tailed Student’s *t*-test; ***P < 0.001. Experiments were performed three times with similar results. Source data are provided as a source data file.

To understand which amino acid residues of HY5 were important for its interaction with BIN2, we screened BIN2-interacting sites in the HY5 LZ region. As shown in Fig. 5a, changing HY5 Leu^137^ (L137) to Asp (named HY5-M) attenuated interaction of HY5 with BIN2 in yeast. In vitro pull-down and LCI assays confirmed the attenuated interaction between HY5-M and BIN2 (Fig. 5b, c). Together with these results, we demonstrated that the L137 site of HY5 is important for its interaction with BIN2.

Next, we chose the promoter of *EXP2*, one of the target genes of HY5^32^, to test transcriptional activation activity of HY5-M. Yeast one-hybrid assays showed that HY5-M protein could bind to the *EXP2*_*pro*_ as well as HY5-WT (Fig. 5d). Protoplast transient expression assays in *Arabidopsis* showed that HY5-M could activate the transcription of *EXP2*_*pro*_*: LUC* at normal levels (Fig. 5e, f). Taken together, L137 of HY5 is not necessary for its transcriptional activation activity.

Because of the attenuated interaction between HY5-M and BIN2, we performed in vitro kinase assays to test whether HY5-M could regulate the kinase activity of BIN2. However, adding HY5-M protein did not obviously increase the phosphorylation level of BZR1 (Fig. 5g), indicating that HY5-M greatly reduced the ability to regulate BIN2 activity in vitro. Consistent with this, the dephosphorylated forms of BZR1 accumulated to higher levels in HY5-M/*hy5* lines than in HY5-WT/*hy5* (Fig. 5h). Likewise, the hypocotyls of HY5-M/*hy5* lines were significantly longer than those of HY5-WT/*hy5* (Fig. 5i).

### HY5 facilitates the autophosphorylation of BIN2 Y200

**Fig. 6:**
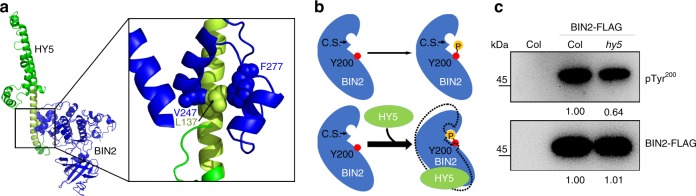
HY5 promotes the autophosphorylation of the BIN2 Y200 residue. **a** (Left) The interaction of HY5 (green) with BIN2 (blue) predicted by computational protein–protein docking. HY5 LZ region, pale green. (Right) Zoom in on the interaction between HY5 and BIN2. Blue sphere, V247 and F277 of BIN2 respectively. Pale green sphere, L137 of HY5. **b** A cartoon diagram showing the intramolecular movement of BIN2 in the presence of HY5. C.S., active catalytic sites. P in yellow ball, phosphate group. Y200 residue of BIN2 is shown as red ball. The dotted lines represent the structural state of BIN2 before binding with HY5. The thickness of long arrows indicates the relative autophosphorylation level of Y200 of BIN2. **c** Immunoblots showing the levels of BIN2 pTyr^200^ in vivo. BIN2-FLAG immunoprecipitated by anti-FLAG antibody were used to detect the pTyr^200^ of BIN2. Numbers under each lane indicate the band intensities of pTyr^200^ or total BIN2-FLAG, respectively. Source data are provided as a source data file.

To gain further insights into the mechanism by which HY5 modulates BIN2 activity, we analyzed the structure of the HY5-BIN2 complex by the computational protein-protein docking method. We found that HY5 interacts with BIN2 through hydrophobic interactions between L137 of HY5 and V247 and F277 of BIN2 (Fig. 6a), in agreement with our finding that L137 of HY5 was important for the HY5–BIN2 interaction.

When we further studied the structure of the HY5–BIN2 complex by coarse-grained functional simulations we found that the amplitude of the functional motion between the N and C domains of BIN2 was much greater in the presence of HY5 (Fig. 6b, Supplementary Movies 1 and 2). It is worth noting that with HY5, the direction of BIN2 motion also changed from twist to open-close, which lead to the intramolecular approach of active catalytic sites of BIN2 to its Y200 residue. Given that the intramolecular phosphorylation of BIN2 Y^200^ residue (pTyr^200^) is critical for its kinase activity^33^ and that the access of Y200 to active catalytic pocket should promote the autophosphorylation of Y200 of BIN2 (Fig. 6b), we speculated that HY5 may affect the level of pTyr^200^ in vivo. Indeed, when we used a specific antibody for detecting pTyr^200^ of BIN2^33^, we found that the lack of HY5 led to a decline in pTyr^200^ level, suggesting that the HY5–BIN2 interaction was important for BIN2 activity.

### Light adjusts HY5-mediated regulation of BIN2 activity

**Fig. 7:**
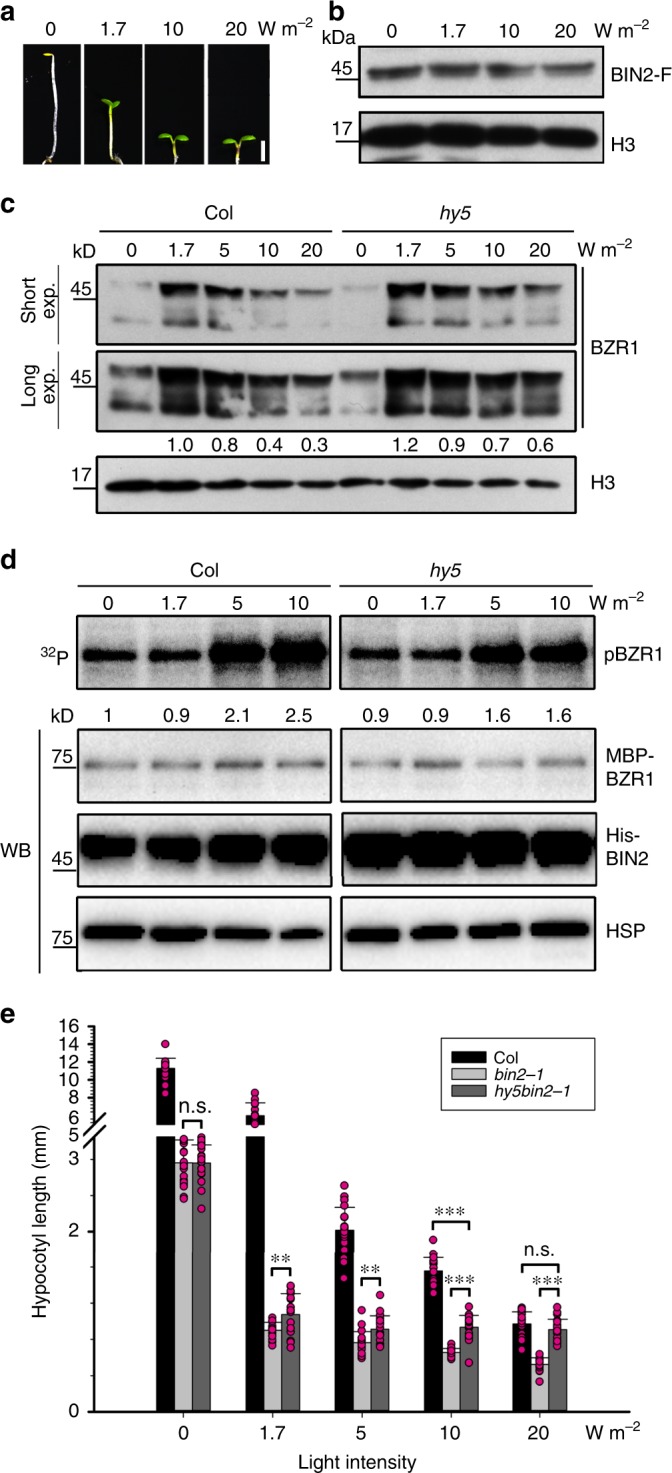
The HY5-BIN2 module regulates hypocotyl elongation subtly in varying light intensities. **a** Morphology of Col grown under different light intensities for 6 days. Scale bar, 1 mm. **b** BIN2-FLAG (BIN2-F) protein levels in transgenic plants that overexpress *BIN2*. Transgenic seedlings were grown for 6 days under the indicated light intensities. H3 was used as a loading control. **c** BZR1 protein levels in Col and *hy5*. Seedlings were grown for 6 days under the indicated light intensities. H3 was used as a loading control. Numbers under lanes indicate relative BZR1 band intensities that were quantified for each panel. **d** Cell-free kinase assay showing BIN2-mediated phosphorylation of BZR1 in the total lysates of Col and *hy5*, which were grown under the indicated light intensities. Numbers under lanes indicated the relative levels of BIN2-mediated phosphorylation of BZR1. HSP was used as a loading control. **e** Hypocotyl lengths of mutants. Seedlings were grown for 6 days under the indicated light intensities. Error bars represent SD, *n* ≥ 20. Asterisks indicate the *P* value of two-tailed Student’s *t*-test; **P < 0.01; ***P < 0.001; n.s., not significant. Experiments were performed three times with similar results. Source data are provided as a source data file.

It has been shown in *Arabidopsis* that increased light intensities result in the gradual accumulation of HY5 proportional to the reduction in hypocotyl length^3^ (Fig. 7a). We therefore determined the effect of varying light intensities on BZR1 phosphorylation and stability regulated by the HY5-BIN2 module. The levels of BIN2-F protein were very similar in the seedlings grown under increasing light intensities (Fig. 7b). We then analyzed the abundance of BZR1 in WT and *hy5* mutant seedlings. Interestingly, as the light intensity increased, the levels of BZR1 protein decreased in WT. The rate of this decrease was obviously slower in *hy5*, suggesting that light intensity regulated BZR1 stability in a partially HY5-dependent manner (Fig. 7c). In agreement with this result, using cell-free kinase assays we found that the BZR1 phosphorylation levels were much higher in WT lysates than in those of *hy5* extracted from seedlings grown under increasing light intensities (Fig. 7d). These results demonstrated that light intensity promoted BIN2-mediated BZR1 phosphorylation and degradation at least in part by controlling HY5 abundance.

To further determine the effect of the HY5-BIN2 module on regulating hypocotyl elongation, we compared the hypocotyl lengths of *bin2-1*, *hy5*, and *hy5bin2-1* seedlings grown under various light intensities. The hypocotyl lengths of *bin2-1* and *hy5bin2-1* were comparable in the dark (Fig. 7e). Under low light (1.7 W m^−^^2^), the hypocotyls of *hy5bin2-1* were slightly longer than *bin2-1*, but still much shorter than wild type. In contrast, under high light (20 W m^−2^), the hypocotyls of *hy5bin2-1* were much longer than *bin2-1*, and were as long as wild type (Fig. 7e). Taken together, HY5 increasingly contributed to BIN2 activity as light intensity increased. Therefore, by responding to varying light intensity, seedlings could adjust hypocotyl elongation by modulating the HY5-mediated regulation of BIN2 activity.

## Discussion

To date, HY5 has been shown to function as a transcription factor that regulates gene expression. Many genes involved in hypocotyl elongation are the targets of HY5, such as LONG HYPOCOTYL IN FAR-RED 1 (HFR1)^8^, FAR-RED ELONGATED HYPOCOTYL 1 (FHY1)^34^, INDOLE-3-ACETIC ACID INDUCIBLE 19 (IAA19)^35^, EXPANSIN 2 (EXP2)^35^. There is no doubt that HY5 is a high level regulator of the transcriptional cascades controlling photomorphogenesis^8^. However, in this study, we revealed that HY5 repressed BR-mediated hypocotyl elongation in a way distinct from its function as a transcription factor. At low light intensities, the abundance of HY5 is limited by COP1-mediated ubiquitination and degradation. BIN2 was at least in part inactive at low HY5 levels, which led to the accumulation of BZR1 and its transcriptional activation of genes promoting hypocotyl elongation. As the light intensity increased, HY5 could accumulate due to the inactivation of COP1, thereby enhancing BIN2 activity through physical interaction. This sequentially promoted BIN2-mediated phosphorylation and degradation of BZR1 to inhibit transcription of genes downstream of BZR1, with the end result of repressing *Arabidopsis* hypocotyl elongation. Moreover, with increasing light intensity, an increasing amount of HY5 protein could bind BIN2 to greater enhance its activity (Supplementary Fig. 8). Therefore, in order to respond to varying light intensities, plants have used the flexible HY5-BIN2-BZR1 cascade to subtly adjust hypocotyl growth.

It has long been uncertain whether light regulates BR signaling or BR regulates light signaling. Restricted by insufficient energy and resources, plants must balance their growth with survival. Therefore, hypocotyl elongation must be precisely regulated, since the coordination of light signaling with signaling by internal hormones (including BR) is important for plant survival and growth. Previous work showed that addition of BL did not affect HY5 stability^27^. In turn, our work showed that HY5 destabilized BZR1 by promoting the kinase activity of BIN2 in the light, indicating BR signaling acted epistatically to light signaling. Recent studies revealed that the light-repressed transcription factors PIF4 and PIF5 bound to the promoter regions of key BR biosynthetic genes to directly promote their expression, thereby inducing BR synthesis^36,37^. Taken together, light signaling may repress BR signaling by inhibiting PIF-induced BR synthesis and promoting HY5-induced BIN2 activity in *Arabidopsis*.

Previously, several key players have been identified in the regulation of BIN2 stability or activity. For instance, BRI1 SUPPRESSOR 1 (BSU1) and HISTONE DEACETYLASE 6 (HDA6) repress BIN2 activity through dephosphorylation and deacetylation of BIN2, respectively^33,38^, while OCTOPUS (OPS) directly represses BIN2 function by sequestering it to the plasma membrane^39^. KINK SUPPRESSED IN bzr1-1D (KIB1) acts as an F-box E3 ubiquitin ligase that promotes the degradation of BIN2^40^. However, little is known about positive regulators promoting BIN2 activity. Our work identified HY5 as one of the positive regulators important for the regulation of BIN2 activity in vivo. It is interesting that we observed the strong effect of HY5 overexpression on the reduction of BZR1 expression while the up-regulation of BZR1 in the *hy5* mutant is relatively mild (Figs. 3 and 4). These data suggest that HY5 may have functional homologs in modulating BIN2-dependent regulation of BZR1. It will be of great interest to define and characterize these factors in the future study.

Previous studies have shown that Y216 in GSK3β is conserved in all GSK3s identified so far, and its phosphorylation is essential for full kinase activity of GSK3s^41,42^. Y216 phosphorylation in GSK3β was an intramolecular autophosphorylation event in mammalian cells^43^, and required the presence of HEAT SHOCK PROTEIN 90 (HSP90)^44^. In this study, we found that, similar to HSP90 in humans, HY5 plays a role in promoting the autophosphorylation of BIN2 Y200, the homolog of GSK3β Y216, to modulate its kinase activity. Our work expands the understanding of regulation of GSK3 kinase activity in general.

## Methods

### Plant materials and growth conditions

The ecotypes of all wild-type *Arabidopsis thaliana* used in this study were Columbia-0 (Col) and Wassilewskija-2 (Ws). The *bin2-1*, *bzr1bes1*, *hy5*, and *bin2-3bil1bil2* mutants were reported previously^17,31,45,46^. Seeds were sterilized with 15% bleach. After 2 days of stratification at 4 °C, seeds were grown on MS medium (pH 5.7) supplemented with 1% sucrose and 0.6% Agar (A1296; Sigma-Aldrich). Six-day-old seedlings grown under cWL were used in this study unless otherwise indicated. For MG132 or BK treatment, seedlings grown in the light were transferred to liquid MS solution supplemented with 30 μM MG132 or 10 μM BK, respectively, for 6 h before harvest.

### Plasmid construction and generation of transgenic lines

To generate the pLacZi-*EXP2*_*pro*_ construct for yeast one-hybrid assays, the subfragment of the *EXP2* promoter^32^ was amplified and inserted into the *Kpn*I/*Xho*I sites of the pLacZi vector^47^.

To generate pB42AD-*HY5* constructs for yeast two-hybrid assays, fragments containing full-length *HY5* coding sequence (CDS) (1-168 aa), and truncated *HY5* CDS including *HY5-N* (1-77 aa), *HY5-C* (78-168 aa), and *HY5-ΔLZ* (deletion of 115-147 aa) were amplified and inserted into the *Eco*RI/*Xho*I sites of the pB42AD vector (Clontech). For the BD-*BIN2*, BD-*BIL1* and BD-*BIL2* constructs, DNA fragments encoding full-length *BIN2*, *BIL1*, and *BIL2* CDS were amplified and inserted into the *Eco*RI/*Bam*HI sites of the pLexA vector (Clontech).

For purification of His-BIN2, GST-HY5, and HY5-His recombinant proteins, the full-length *BIN2* CDS fragment was inserted into the *Eco*RI/*Sal*I sites of the pET28a vector, full-length and truncated *HY5* fragments were inserted into the *Eco*RI/*Xho*I sites of the pGEX4T-1 vector or *Nco*I/*Not*I sites of the pET28a vector. The *MBP-PIF3* construct was reported previously^47^.

To generate *HY5-nLUC* constructs, full-length and truncated *HY5* fragments were cloned into the *Kpn*I/*Sal*I sites of the pCAMBIA1300-*nLUC* vector. The *cLUC-BIN2*, *cLUC-BIL1*, and *cLUC-BIL2* constructs were reported previously^48^.

To generate the *EXP2*_*pro*_*-LUC* construct, a DNA fragment with 1021 bp upstream of the ATG together with 49 bp downstream of the ATG of *EXP2* was amplified and cloned into the *Kpn*I/*Nco*I sites of the pGreenII 0800-*LUC* vector^49^.

The *bzr1bes1* double mutant was generated by egg cell-specific promoter-controlled CRISPR/Cas9^31^, and then crossed with *hy5* to get a *hy5bzr1bes1* triple mutant. To generate transgenic plants overexpressing 3×HA-tagged HY5, the promoter of *Arabidopsis UBQ10* was amplified from genomic DNA^50^, then the *UBQ10*_*pro*_*:3×HA-HY5-3’UTR-OCS*_*terminator*_ DNA fragment was obtained by overlap extension PCR, and cloned into the *Eco*RI/*Kpn*I sites of the pCAMBIA1300 vector. The *UBQ10*_*pro*_*:3×HA-HY5*^*L137D*^ fragment was amplified by primer-based site-directed mutagenesis and then also cloned into the *Eco*RI/*Kpn*I sites of the pCAMBIA1300 vector. For stable transformation, *Agrobacterium tumefaciens* strain GV3101 carrying the construct was then used to transform the transgenes into Col, Ws, and other related mutants using the floral dip method^51^. Homozygous lines were screened based on hygromycin resistance. Generation of *BIN2-FLAG* transgenic plants was reported previously^48^. All the cloning and genotyping primers are listed in Supplementary Table 1.

### Western blot and antibodies

For protein extraction, seedlings were frozen in liquid nitrogen, ground into powder, then resuspended in 2× SDS buffer (0.125 M Tris-HCl [pH 6.8], 4% SDS, 20% glycerol, 1× cocktail of protease and phosphatase inhibitors, 1 mM PMSF). Samples were heated for 10 min at 65 °C, then centrifuged at 13,000 × *g* for 10 min at room temperature. The supernatants were transferred into new tubes, and the total protein concentrations were determined by the BCA method. Equal amounts of total proteins were separated in 10% sodium dodecyl sulfate (SDS)-polyacrylamide gels and then transferred onto PVDF membranes. The subsequent immunoblots were performed as previously described^52^. Antibodies used in this study were anti-HY5 (A gift from Rongcheng Lin’s lab, 1:1000 dilution), anti-BZR1 (A gift from Jianming Li’s lab, 1:1000 dilution), anti-Histone H3 (05-499, Millipore, 1:1000 dilution), anti-HSP (AbM51099-31-PU, Beijing Protein Innovation, 1:5000 dilution), anti-HA (H9658-.2 ML, Sigma-Aldrich, 1:2000 dilution), anti-Flag (F3165-.2MG, Sigma-Aldrich, 1:2000 dilution), Anti-phospho-GSK3 (Tyr279/Tyr216) (05-413, Millipore, 1:1000 dilution), anti-MBP (#E8031S, New England Biolabs, 1:5000 dilution), anti-His (H1029-.2ML, Sigma-Aldrich, 1:2000 dilution), and anti-GST (#2625, Cell Signaling Technology, 1:1000 dilution).

### Yeast one-hybrid and two-hybrid assays

Yeast one-hybrid assays were performed as described previously^11^. Briefly, pB42AD-*HY5* (effector) and pLacZi-*EXP2*_*pro*_ (reporter) were co-transformed into yeast strain EGY48, the transformants were plated on minimal synthetic defined (SD) base supplemented with the −Ura/−Trp dropout (DO) mix and X-gal (5-bromo-4-chloro-3-indolyl-β-d-galactopyranoside) for blue color development. Yeast two-hybrid assays based on the LexA system were performed according to the standard protocol (Clontech). The yeast strain used in this study was EGY48 containing p8oplacZ vector.

### In vitro pull-down assays

For in vitro pull-down assays, 1 μg GST-HY5 and 1 μg His-BIN2 proteins were incubated in 1 ml binding buffer (25 mM Tris-Cl [pH 7.5], 100 mM NaCl, 0.1% NP40) at 4 °C for 1 h. Ten microliters GST beads pre-washed with PBS were added into the binding buffer and incubated with the proteins for another 1 h. After that, the beads were washed three times with washing buffer (25 mM Tris-Cl [pH 7.5], 500 mM NaCl) at 4 °C. GST beads were heated for 5 min in 1× SDS loading buffer at 100 °C. The eluted proteins were then analyzed by immuno-blotting using anti-GST and anti-His antibodies.

### Co-IP assays

For in vivo Co-IP assays, 0.5 g seedlings were frozen in liquid nitrogen, ground into powder, and added into lysis buffer (25 mM Tris-HCl [pH 7.5], 150 mM NaCl, 1 mM EDTA, 10% glycerol, 0.5% Tween-20). Samples were centrifuged at 15,000 × *g* for 10 min, and then total protein concentrations of the supernatants were determined by the Bradford method. One milligram total proteins were incubated with 10 μl anti-FLAG beads in 1 ml lysis buffer for 3 h at 4 °C. After that, the beads were washed three times with washing buffer (25 mM Tris-Cl [pH 7.5], 150 mM NaCl, 0.1% Tween-20) at 4 °C. The proteins were eluted from the beads with the addition of 3× FLAG peptide (F4799, Sigma-Aldrich). Eluted proteins were analyzed by immuno-blotting using anti-BZR1, anti-FLAG. and anti-HSP antibodies.

For semi-in vivo Co-IP assays, total proteins of Col or BIN2-FLAG seedlings were extracted using the lysis buffer (25 mM Tris-HCl [pH 7.5], 150 mM NaCl, 1 mM EDTA, 10% glycerol, 0.1% NP40). Five hundred micrograms total proteins were incubated with 8 μg GST-HY5 protein in 0.5 ml lysis buffer for 2 h at 4 °C, then 10 μl anti-FLAG beads were added and incubated with the mixture for three more hours at 4 °C. After that, the beads were washed three times with 25 column volumes of washing buffer (25 mM Tris-Cl [pH 7.5], 500 mM NaCl, 1 mM EDTA, 10% glycerol). The proteins were eluted with 3× FLAG peptide and analyzed by immuno-blotting using anti-GST, anti-FLAG, and anti-HSP antibodies.

### LCI assay

The LCI assays were performed as previously described with some modifications^53^. In brief, GV3101 colonies containing different constructs were inoculated into 5 ml LB medium supplemented with kanamycin, and grown at 28 °C for 16 h. In all, 0.1 ml of the cultures were transferred to 5 ml LB supplemented with 10 mM MES (pH 5.6) and 40 μM acetosyringone. Bacteria were grown at 28 °C for 16 h, and harvested by centrifugation. The bacteria were resuspended in buffer containing 10 mM MES (pH 5.6), 10 mM MgCl_2_, and 100 μM acetosyringone. Related bacteria were mixed and each bacterium had a final concentration of OD_600_ = 0.5. The bacteria were kept at room temperature for 3–5 h without shaking. Infiltration was performed with a 2-ml syringe without needle. The plants were subsequently kept away from light for 12 h, and then grown in the light for 2 days before the analysis. The luciferase signals were analyzed with Night SHADE LB 985 (Berthold Technologies).

### Protoplast transient expression assays

Protoplasts from *Arabidopsis* mesophyll cells were prepared and transformed as described previously^54^. The effector plasmid *UBQ10*_*pro*_*:HA-HY5*, reporter *EXP2*_*pro*_*:LUC*, and control *35S:REN* were co-transformed into protoplasts and incubated under weak light for 12 h. Then the protoplasts were harvested, and the luminescent signals of LUC and REN were detected with the Dual-Glo^®^ Luciferase Assay System (E2920, Promega). The *LUC* expression levels were quantified as the ratio of LUC/REN enzyme activities.

### Quantitative real-time PCR

Total RNA was extracted from seedlings using RNeasy Plant Mini Kits (74904, QIAGEN). One microgram of total RNA was incubated with DNase I at 37 °C to remove the genomic DNA, and then the prepared RNA was used as a template for RevertAid Reverse Transcriptase (EP0442, Thermo Scientific) to generate first-strand cDNA. qPCR was performed using a 7500 Fast Real-Time PCR System (Applied Biosystems). The CT values were used to calculate the expression levels of different genes normalized to *PP2A*. All the RT-qPCR primers are listed in Supplementary Table 1.

### In vitro kinase assays

In vitro and cell-free kinase assays were performed as previously described with some modifications^55^. For in vitro kinase assays, 50 ng His-BIN2 were pre-mixed with HY5-His, the mole ratio of BIN2/HY5 ranging from 1:1 to 1:8 as indicated, then 1 μg MBP-BZR1 was incubated with the mixture in 20 μl kinase buffer (20 mM Tris-HCl [pH 7.5], 100 mM NaCl, 12 mM MgCl_2_, 0.1 mM ATP, 0.2 μCi [γ-^32^P] ATP) at 30 °C for 30 min. For the cell-free kinase assays, total proteins were extracted from Col and *hy5*, respectively, in the buffer (20 mM Tris-HCl [pH 7.5], 100 mM NaCl, 12 mM MgCl_2_) containing 1× cocktail of protease and phosphatase inhibitors, and 1 mM PMSF. Five micrograms total proteins were incubated with 1 μg MBP-BZR1 and 50 ng His-BIN2 in 20 μl kinase buffer containing 1× cocktail of protease and phosphatase inhibitors. After incubation at 30 °C for 30 min, the reactions were terminated in 1× SDS loading buffer, and samples were separated in 10% SDS-polyacrylamide gels. ^32^P signals were detected with Typhoon FLA7000 (GE Healthcare).

### Computational methods

The monomeric HY5 and BIN2 proteins were modeled using JACKAL^56^ and ITASSER programs^57^. The models with the highest scores were chosen as the results. The predicted structure was then minimized using molecular dynamics simulation package Gromacs 4.5^58^.

The complex formed by the interaction between HY5 and BIN2 was predicted by the docking method HoDock^59^, which incorporates an initial rigid docking and a refined semi-flexible docking. A total of 200,000 complex structures were generated and scored to select the final correct complex structure model. The docked complex model was also minimized using the molecular dynamics simulation package Gromacs 4.5. The interface contact residues were calculated by the Cartesian coordinates of non-hydrogen heavy atoms C, N, and O. Those residues with at least one pair of heavy atoms within 4 Å were denoted as interface contact residues.

The minimized monomeric BIN2 and complex BIN2-HY5 structures were used to simulate the functional motions by a coarse-grained anisotropic network model^60^. All data were depicted and presented using PyMOL software. The movies were created using the PyMOL frame pictures and Matlab script.

### Primer sequences

The sequences for all primers used in this study are listed in Supplementary Table 1.

### Reporting summary

Further information on research design is available in the Nature Research Reporting Summary linked to this article.

## Supplementary information

Supplementary Information

Description of Additional Supplementary Files

Supplementary Movie 1

Supplementary Movie 2

Reporting Summary

## Data Availability

The source data generated and analyzed in this study are provided as a Source Data file. All relevant data are available from the corresponding authors upon request. There are no restrictions on data availability.

## Source Data

Source Data
