# Supplementary material for: Modulation of BIN2 kinase activity by HY5 controls hypocotyl elongation in the light
Source: Nat Commun. 2020 Mar 27;11:1592. doi: 10.1038/s41467-020-15394-7 (PMC7101348; doi:10.1038/s41467-020-15394-7)
Supplement: Supplementary file 1 — Supplementary Information [file 41467_2020_15394_MOESM1_ESM.pdf]

Modulation of BIN2 kinase activity by HY5 controls hypocotyl elongation in the light

Li et al.

Supplementary Information

This document contains Supplementary Fig. 1~8 with figure legends, Supplementary Table 1 (the primer list) and Description of Additional Supplementary Files.

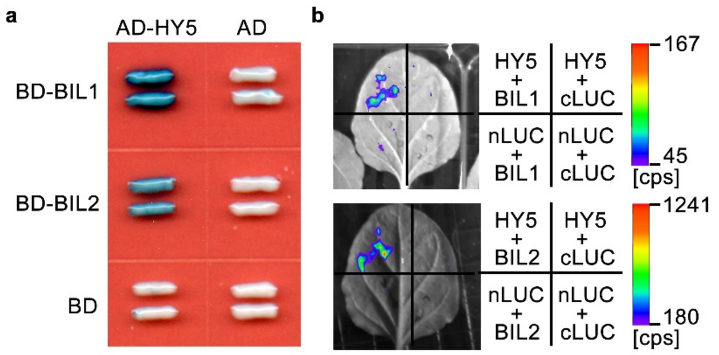

Supplementary Fig. 1 The interactions between HY5 and homologs of BIN2, BIL1 and BIL2. **a** HY5 interacted with BIL1 and BIL2 in yeast. **b** LCI assay showing the interaction of HY5 and BIL1, BIL2 respectively in *N. tabacum* leaves. cps, counts per second.

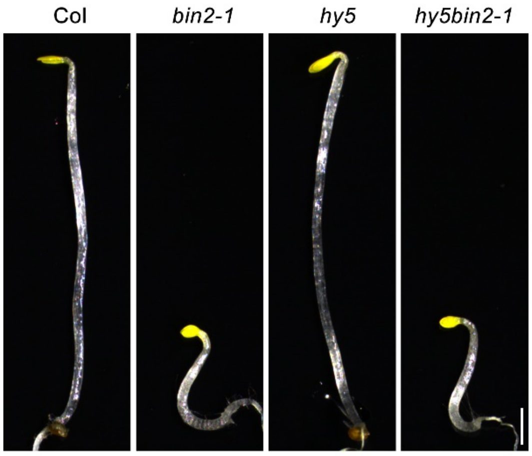

Supplementary Fig. 2 Morphology of *hy5bin2-1* in the dark. Seedlings were grown in the dark for 5 days. Bar, 1mm.

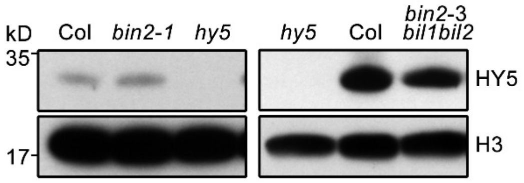

Supplementary Fig. 3 Protein levels of HY5 in *bin2-1* and *bin2-3bil1bil2* in the light.

H3 was used as a loading control. Source data are provided as a source data file.

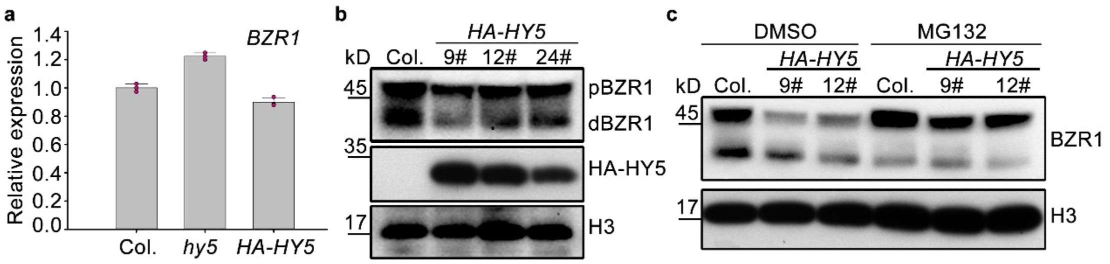

Supplementary Fig. 4 HY5 destabilizes BZR1 via the 26S proteasomal pathway in the light. **a** RT-qPCR showing relative expression of *BZR1* in Col and *HA-HY5* seedlings. The relative expression levels were normalized to *PP2A*. Error bars represent SD, n=3. RT-qPCR was performed three times with similar results. **b** Protein levels of BZR1 in independent *HA-HY5* lines. H3 was used as a loading control. **c** Protein levels of BZR1 in *HA-HY5* lines treated with 30 μM MG132. H3 was used as a loading control. Source data are provided as a source data file.

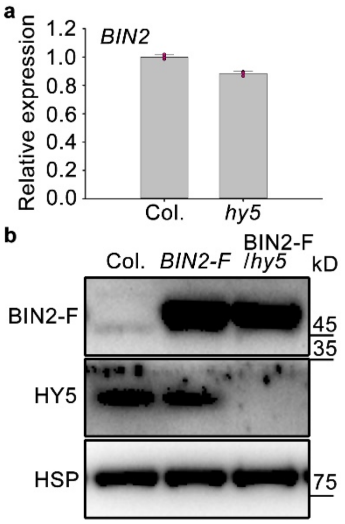

Supplementary Fig. 5 The mRNA and protein levels of BIN2 in *hy5*. **a** RT-qPCR showing relative expression of *BIN2* in Col and *hy5* seedlings. The relative expression levels were normalized to *PP2A*. Error bars represent SD, n=3. RT-qPCR was performed three times with similar results. **b** The protein level of BIN2-FLAG (BIN2-F) in *hy5*. HSP was used as a loading control. Source data are provided as a source data file.

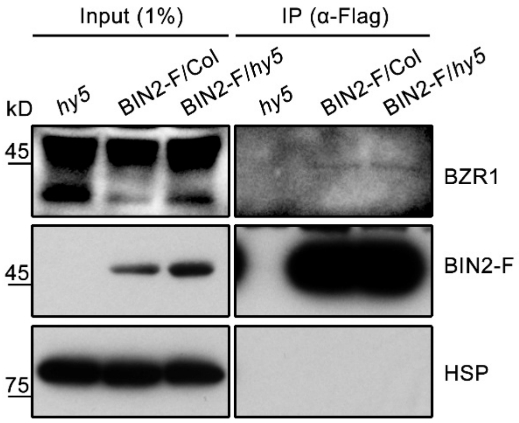

Supplementary Fig. 6 The association of BIN2 and BZR1 in the absence of HY5 assayed by coimmunoprecipitation (Co-IP) in vivo. Antibody to FLAG was used to precipitate proteins, and the pellets were analyzed by western blots using antibodies to FLAG and BZR1 respectively. HSP was used as a loading control. Source data are provided as a source data file.

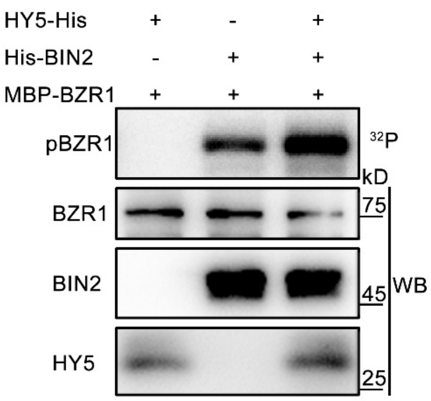

Supplementary Fig. 7 HY5 promotes BIN2-mediated BZR1 phosphorylation in vitro. ^{32}P, autoradiography by [γ-^{32}P] ATP-labeled proteins. WB, western blot of proteins used in the kinase assay. Source data are provided as a source data file.

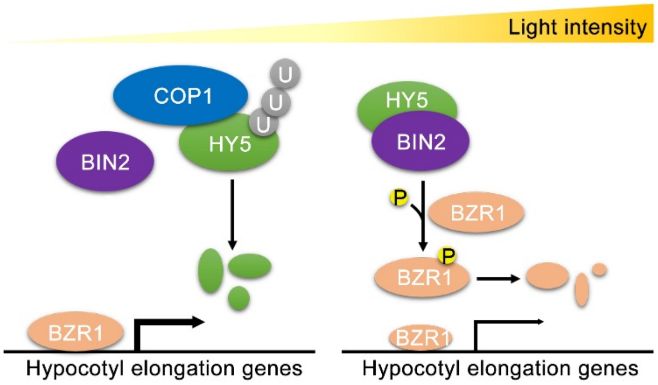

Supplementary Fig. 8 A working model of how the HY5-BIN2 module represses hypocotyl elongation in varying light intensities. U, ubiquitin protein. P, phosphate group. See the text for details.

Supplementary Table 1. The sequences of all primers used in this study.

Primer name	Sequence
**For cloning**	
AD-HY5-F / GST-HY5-F	CGGAATTCATGCAGGAACAAGCGAC
AD-HY5-R / GST-HY5-R	CAACTCGAGTCAAAGGCTTGCATCAG
AD-HY5-N77-R	CAACTCGAGTCATTGACTTTCTCCGACAGTC
AD-HY5-C91-F	CGGAATTCAGGAAGCGAGGGAGGACA
AD-HY5-ΔLZ-MF	AAGGCTTACTTGAGCCTGAAGAACACAACA
AD-HY5-ΔLZ-MR	TGTTGTGTTCTTCAGGCTCAAGTAAGCCTT
AD-HY5-ΔBr-MF	GTCGGAGAAAGTCAAGAGTTGGAAAACAGA
AD-HY5-ΔBr-MR	TCTGTTTTCCAACTCTTGACTTTCTCCGAC
GST-HY5-N77-R	CAACGTCGACTCATTGACTTTCTCCGAC
GST-HY5-C91-F	CTGGATCCAGGAAGCGAGGGAGGA
BD-BIN2-F	CGGAATTCATGGCTGATGATAAGGA
BD-BIN2-R	CGGGATCCTTAAGTTCCAGATTGA
BD-BIL1-F	CGGAATTCATGACTTCGATACCATT
BD-BIL1-R	CGGGATCCCTAGGGTCCAGCTTGAA
BD-BIL2-F	CGGAATTCATGGCCTCATTACCATT
BD-BIL2-R	CGGGATCCTTAACTGTTTTGTAATCC
HY5-His-F	CATGCCATGGGCATGCAGGAACAAGCGA
HY5-His-R	ATAAGAATGCGGCCGCAAGGCTTGCATCAGC
UBQ-HY5-F	CGGAATTCGTCGACGAGTCAGTAATAA

UBQ-HY5-R	GGGGATCCTATCATAGAACAAAAATTAGC
HY5-nLUC-F	CTGGTACCATGCAGGAACAAGCGA
HY5-nLUC-R	ACGCGTCGACAAGGCTTGCATCAGC
HY5-mutation-F	AGCGACTCTCTACTGATCAGAACGAGAACC
HY5-mutation-R	GGTTCTCGTTCTGATCAGTAGAGAGTCGCT
HY5-N77-nLUC-R	ACGCGTCGACTTGACTTTCTCCGAC
HY5-C91-nLUC-F	CTGGTACCATGAGGAAGCGAGGGAGGA
cLUC-BIN2-F	CTGGTACCATGGCTGATGATAAGG
cLUC-BIN2-R	ACGCGTCGACTTAAGTTCCAGATTGA
EXP2pro-LacZ-F	GGGGTACCCCGATTAATTTTATCAAATG
EXP2pro-LacZ-R	CCGCTCGAGCACATATTTTTTTAGAGAAA
EXP2pro-LUC-F	CGGGGTACCTATCAAAATTTCCTGGTGTGG
EXP2pro-LUC-R	CATGCCATGGGGGTGCATAGTGAAAGAAACA
**For generating *bzr1bes1* mutant via CRISPR system**	
BZR1-sgRNA1-F	GATTGAGAAAGGGAGAATAATCGG
BZR1-sgRNA1-R	AAACCCGATTATTCTCCCTTTCTC
BZR1-sgRNA2-F	GATTGGTGCAGAAACCGCATAGAA
BZR1-sgRNA2-R	AAACTTCTATGCGGTTTCTGCACC
BES1-sgRNA1-F	GATTGCGGCGGAGAAGAGCTGTTG
BES1-sgRNA1-R	AAACCAACAGCTCTTCTCCGCCGC
BES1-sgRNA2-F	GATTGAAGTTGGGGATGACACTGG
BES1-sgRNA2-R	AAACCCAGTGTCATCCCCAACTTC

**For genotyping**	
hy5-genotype-F	CCAAGTTCTTCTGTAAATCCCAAGT
hy5-genotype-R	AGGAGATCAAAGGCTTGCATCA
LBb1.3	ATTTTGCCGATTTCGGAAC
bzr1-crispr-seq-F	ACTTCGGATGGAGCTACGTC
bzr1-crispr-seq-R	AGAGAATGGCTGTTGTTGTG
bes1-crispr-seq-F	GCAGCGATGGCGACGAGGAG
bes1-crispr-seq-R	GCATAAAACGGGTAGTTCAA
**For RT-qPCR**	
RT-SAUR15-F	GTGTCTTGTACCAAAAAAAGG
RT-SAUR15-R	ACCAACCAGAATAATGAGTGT
RT-XTH18-F	TCCCGCAAATTCCTCATGGT
RT-XTH18-R	ATCATCTTACACAAACACCGCA
RT-PMEPCRB-F	ACCACTTTCAACTCCGCCAC
RT-PMEPCRB-R	GGTTCTGCATCGGTTGTCTTG
RT-HFR1-F	TGTCACAAGACGGACAAGGT
RT-HFR1-R	GTCAGCATGTGGTTGTGCAT
RT-BIN2-F	ACAAAAGGATGCCCCCAGAA
RT-BIN2-R	TGAAGTTGAAGAGAGGCGGG
RT-BZR1-F	CAACTAGGCAAACCCAAATG
RT-BZR1-R	TCTAACACTCCAATGCTTCC
RT-PP2A-F	TTCGTATCGGTGGTTCTTCTCC

RT-PP2A-R	ACGAACTTTCAGTGCTACCAA

Description of Additional Supplementary Files

Supplementary Movie 1 The functional motion amplitude and direction of monomer BIN2. The structure of BIN2 was shown in stick with blue color. Balls presented the key residues of BIN2, Y200 was shown with green color, the other residues involving in ATP binding (yellow balls) and substrate binding (red balls) formed the catalytic region of BIN2 kinase.

Supplementary Movie 2 The functional motion amplitude and direction of BIN2 after binding with HY5. The structure of BIN2 was shown in stick with blue color, while HY5 was green. Balls presented the key residues of BIN2, Y200 was shown with green color, the other residues involving in ATP binding (yellow balls) and substrate binding (red balls) formed the catalytic region of BIN2 kinase.
