# Supplementary material for: Modulation of BIN2 kinase activity by HY5 controls hypocotyl elongation in the light
Source: Nat Commun. 2020 Mar 27;11:1592. doi: 10.1038/s41467-020-15394-7 (PMC7101348; doi:10.1038/s41467-020-15394-7)
Supplement: Supplementary file 2 — Description of Additional Supplementary Files [file 41467_2020_15394_MOESM2_ESM.pdf]

Description of Additional Supplementary Files

File Name: Supplementary Movie 1
Description: The functional motion amplitude and direction of monomer BIN2. The structure of BIN2 was shown in stick with blue color. Balls presented the key residues of BIN2, Y200 was shown with green color, the other residues involving in ATP binding (yellow balls) and substrate binding (red balls) formed the catalytic region of BIN2 kinase.

File Name: Supplementary Movie 2
Description: The functional motion amplitude and direction of BIN2 after binding with HY5. The structure of BIN2 was shown in stick with blue color, while HY5 was green. Balls presented the key residues of BIN2, Y200 was shown with green color, the other residues involving in ATP binding (yellow balls) and substrate binding (red balls) formed the catalytic region of BIN2 kinase.
