# Supplementary material for: Modulation of BIN2 kinase activity by HY5 controls hypocotyl elongation in the light
Source: Nat Commun. 2020 Mar 27;11:1592. doi: 10.1038/s41467-020-15394-7 (PMC7101348; doi:10.1038/s41467-020-15394-7)
Supplement: Supplementary file 5 — Reporting Summary [file 41467_2020_15394_MOESM5_ESM.pdf]

nature research

Corresponding author(s): Xing Wang Deng

Last updated by author(s): Feb 25, 2020

Reporting Summary

Nature Research wishes to improve the reproducibility of the work that we publish. This form provides structure for consistency and transparency in reporting. For further information on Nature Research policies, see Authors & Referees and the Editorial Policy Checklist .

Statistics

For all statistical analyses, confirm that the following items are present in the figure legend, table legend, main text, or Methods section.

n/a	Confirmed	
☐	☒	The exact sample size (*n*) for each experimental group/condition, given as a discrete number and unit of measurement
☐	☒	A statement on whether measurements were taken from distinct samples or whether the same sample was measured repeatedly
☒	☐	The statistical test(s) used AND whether they are one- or two-sided<br>*Only common tests should be described solely by name; describe more complex techniques in the Methods section.*
☒	☐	A description of all covariates tested
☒	☐	A description of any assumptions or corrections, such as tests of normality and adjustment for multiple comparisons
☒	☐	A full description of the statistical parameters including central tendency (e.g. means) or other basic estimates (e.g. regression coefficient) AND variation (e.g. standard deviation) or associated estimates of uncertainty (e.g. confidence intervals)
☒	☐	For null hypothesis testing, the test statistic (e.g. *F*, *t*, *r*) with confidence intervals, effect sizes, degrees of freedom and *P* value noted<br>*Give P values as exact values whenever suitable.*
☒	☐	For Bayesian analysis, information on the choice of priors and Markov chain Monte Carlo settings
☒	☐	For hierarchical and complex designs, identification of the appropriate level for tests and full reporting of outcomes
☒	☐	Estimates of effect sizes (e.g. Cohen's *d*, Pearson's *r*), indicating how they were calculated

Our web collection on statistics for biologists contains articles on many of the points above.

Software and code

Policy information about availability of computer code

Data collection	The Real-time PCR cycler (7500 Fast Real-Time PCR System, Applied Biosystems) was used to quantitatively measure mRNA level; ChemiDoc XRS+ (Bio-Rad) was used for chemiluminescence detection during western blot. Typhoon FLA7000 (GE Healthcare) was used to detect 32P signals; Night SHADE LB 985 (Berthold Technologies) was used to detect luciferase signals; The Image J (https://imagej.nih.gov/ij/) was used to measure the hypocotyl length of Arabidopsis seedlings.
Data analysis	The SigmaPlot was used to analyze hypocotyl elongation of Arabidopsis seedlings; The Microsoft Excel was used to analyze qRT-PCR results and determine statistical significance based on the two-tailed t-Test; The targets of CRISPR/Cas9 were selected with suggestion of CRISPR-PLANT web program (http://crispr.hzau.edu.cn/cgi-bin/CRISPR/CRISPR).

For manuscripts utilizing custom algorithms or software that are central to the research but not yet described in published literature, software must be made available to editors/reviewers. We strongly encourage code deposition in a community repository (e.g. GitHub). See the Nature Research guidelines for submitting code & software for further information.

Data

Policy information about availability of data

All manuscripts must include a data availability statement. This statement should provide the following information, where applicable:

- Accession codes, unique identifiers, or web links for publicly available datasets
- A list of figures that have associated raw data
- A description of any restrictions on data availability

All data are available from the corresponding authors upon a reasonable request.

Field-specific reporting

Please select the one below that is the best fit for your research. If you are not sure, read the appropriate sections before making your selection.

☒ Life sciences ☐ Behavioural & social sciences ☐ Ecological, evolutionary & environmental sciences

For a reference copy of the document with all sections, see nature.com/documents/nr-reporting-summary-flat.pdf

Life sciences study design

All studies must disclose on these points even when the disclosure is negative.

Sample size	20 seedlings for each genotype were collected for hypocotyl length measurements. No statistical method was used to predetermine sample sizes.
Data exclusions	No data were excluded from analyses.
Replication	Experiments were performed three times with similar results.
Randomization	All samples were arranged randomly into experimental groups.
Blinding	Animal experiments were not performed in this study, thus the investigators were not blinded to the experiments reported in this study.

Reporting for specific materials, systems and methods

We require information from authors about some types of materials, experimental systems and methods used in many studies. Here, indicate whether each material, system or method listed is relevant to your study. If you are not sure if a list item applies to your research, read the appropriate section before selecting a response.

Materials & experimental systems

n/a	Involved in the study	
☐	☒	Antibodies
☒	☐	Eukaryotic cell lines
☒	☐	Palaeontology
☒	☐	Animals and other organisms
☒	☐	Human research participants
☒	☐	Clinical data

Methods

n/a	Involved in the study	
☒	☐	ChIP-seq
☒	☐	Flow cytometry
☒	☐	MRI-based neuroimaging

Antibodies

Antibodies used	Anti-HY5, anti-BZR1 are custom-made antibodies. Other antibodies used in this study are commercially available, including anti-Histone H3 (05-499, Millipore), anti-HSP (AbM51099-31-PU, Beijing Protein Innovation), anti-HA (H9658-.2ML, Sigma-Aldrich), anti-Flag (F3165-.2MG, Sigma-Aldrich), anti-MBP (#E8031S, New England Biolabs), anti-His (H1029-.2ML, Sigma-Aldrich) and anti-GST (#2625, Cell Signaling Technology).
Validation	The anti-HY5 antibody was identified using hy5 mutant as a negative control in the study. The anti-BZR1 antibody was previously validated (Yan, et al., 2009, Plant Physiol.; Tang, et al., 2010, Nat. Cell Biol.).
